# Leveraging Large Data, Statistics, and Machine Learning to Predict the Emergence of Resistant *E. coli* Infections

**DOI:** 10.3390/pharmacy12020053

**Published:** 2024-03-22

**Authors:** Rim Hur, Stephine Golik, Yifan She

**Affiliations:** 1 Department of Inpatient Pharmacy, Kaiser Permanente, One Kaiser Plaza, Oakland, CA 94612, USA; 2 Haas School of Business, University of California, Berkeley, CA 94720, USA; 3 School of Pharmacy, University of California, San Francisco, CA 94143, USA; 4 Thomas J. Long School of Pharmacy, University of the Pacific, Stockton, CA 95211, USA

**Keywords:** time series models, ARIMA, neural networks, random forest algorithm, antimicrobial resistance, antibiotic resistance, length of stay (LOS), antimicrobial stewardship programs (ASPs)

## Abstract

Drug-resistant Gram-negative bacterial infections, on average, increase the length of stay (LOS) in U.S. hospitals by 5 days, translating to approximately $15,000 per patient. We used statistical and machine-learning models to explore the relationship between antibiotic usage and antibiotic resistance over time and to predict the clinical and financial costs associated with resistant *E. coli* infections. We acquired data on antibiotic utilization and the resistance/sensitivity of 4776 microbial cultures at a Kaiser Permanente facility from April 2013 to December 2019. The ARIMA (autoregressive integrated moving average), neural networks, and random forest time series algorithms were employed to model antibiotic resistance trends. The models’ performance was evaluated using mean absolute error (MAE) and root mean squared error (RMSE). The best performing model was then used to predict antibiotic resistance rates for the year 2020. The ARIMA model with cefazolin, followed by the one with cephalexin, provided the lowest RMSE and MAE values without signs of overfitting across training and test datasets. The study showed that reducing cefazolin usage could decrease the rate of resistant *E. coli* infections. Although piperacillin/tazobactam did not perform as well as cefazolin in our time series models, it performed reasonably well and, due to its broad spectrum, might be a practical target for interventions in antimicrobial stewardship programs (ASPs), at least for this particular facility. While a more generalized model could be developed with data from multiple facilities, this study acts as a framework for ASP clinicians to adopt statistical and machine-learning approaches, using region-specific data to make effective interventions.

## 1. Introduction

According to the U.S. Centers for Disease Control and Prevention (CDC), almost 30% of antibiotics prescribed in acute care hospitals are unnecessary or suboptimal. Inappropriate use of antibiotics increases the risk of resistance, adverse drug events, and the emergence of secondary infections [1]. Additionally, evidence shows that patients are often discharged from hospitals with excess antibiotics, leading to unnecessary use of antibiotics and the emergence of antibiotic resistance [2].

Research indicates that antibiotic usage is correlated with the emergence of antibiotic resistance [3,4,5,6,7,8,9,10,11]. For example, a statistically significant correlation was shown between consumption of antibiotics and resistance rates of Pseudomonas [9]. Ryu et al. [12] showed that the use of beta-lactam/beta-lactamase inhibitor antibiotics such as ampicillin/sulbactam and piperacillin/tazobactam is significantly correlated with increased rates of piperacillin/tazobactam-resistant Klebsiella pneumoniae [12]. Additionally, broad spectrum antibiotics have been shown to correlate with the emergence of multi-drug resistant infections [6,13]. 

Resistant infections pose not only clinical challenges, but a financial burden. Hospital operational costs are increased by patients’ prolonged hospital stays, which are frequently required to treat resistant diseases. More specifically, a resistant *E. coli* infection on average increases the LOS for a patient in the United States by 5 days [9]; this observation can be extrapolated to other resistant Gram-negative infections such as Klebsiella and Pseudomonas [9]. Since overnight hospital stays cost an average of $2883 per day in the U.S. [14], a resistant Gram-negative infection can cost approximately $14,415, just for the extended LOS alone. 

In addition to reporting correlation coefficients between antibiotic usage and antibiotic resistance, scientists have used time series models such as the Box–Jenkins method to document the development of antibiotic resistance over time as a result of antibiotic usage [11,15,16,17,18]. At the same time, machine learning techniques such as artificial neural networks (ANNs) and random forest algorithms are becoming an integral forecasting tool in pharmaceutical and healthcare-related research [19,20]. Those ANNs are being applied in the pharmaceutical arena to predict how novel drug molecules would behave in the human body [21] and to predict antibiotic resistance [22,23,24]. Michael Kane, a researcher and a professor at Yale University, showed that the performance of random forest time series algorithms can outperform existing time series models for predicting infectious disease outbreaks [19]. 

Statistical and machine learning models can be used to better understand the relationship between antibiotic usage and the development of antibiotic resistance over time, and to predict the clinical and financial cost of using culpable antibiotics. The CDC and the Healthcare Infection Control Practices Advisory Committee (HICPAC) recommend implementing ASP programs and guidelines for antibiotic use to ensure appropriate selection, dose, route of administration, and duration of therapy [25]. This study was devised and executed to kickstart and encourage the application of statistical and machine learning approaches to region-specific data. The aim is to guide and support local hospital ASPs in both acute care and discharge settings. 

## 2. Materials and Methods

### 2.1. Study Aim, Design, and Setting

The aim of the study is to identify the antibiotics that are most strongly associated with resistant *E. coli* infections and to explore how changes in antibiotic usage might impact both resistance rates and the associated costs. This was a data only, observational study for quality improvement for Kaiser Permanente Vacaville Medical Center, which is a 152-bed level 2 trauma center located in Vacaville, California. The study included all microbial cultures of *E. coli* from all sources of infection (abscess, blood, urine, and wounds) at the Kaiser Permanente Vacaville Medical Center. We also included total antibiotic utilization at the facility (reported as days of therapy per 1000 patient days) of cephalosporins, beta lactams, fluoroquinolones, and aminoglycosides (oral and intravenous routes). Utilizing data spanning 1 April 2013 to 31 December 2018, we trained our statistical and machine learning algorithms. Subsequently, data from the year 2019 (1 January 2019 to 31 December 2019) enabled us to test the accuracy of our models and to generate estimates of future antibiotic resistance at KP Vacaville Medical Center. This observational approach allows us to identify patterns and associations, though it is critical to understand that these do not necessarily imply causation between specific antibiotic use and the emergence of resistance.

#### 2.1.1. Study Population 

The study population included 4776 microbial cultures that were identified through Kaiser Permanente’s electronic health record. The inclusion and exclusion criteria of the study are tabulated in Table 1. 

#### 2.1.2. Study Endpoints

The primary outcome of this study was to identify the target antibiotic used by Kaiser Permanente Vacaville that led to increased antibiotic resistance in *E. coli*, indicated by resistance to ceftriaxone. The secondary outcome was resistance rate prediction for the year 2020.

### 2.2. Input Feature Selection for Time-Series Models

The antibiotics selected in this section (i.e., Section 2.2 Input Feature Selection for Time-Series Models) by the correlation analysis, stepwise linear regression, and recursive feature elimination process were used as exogenous regressors to the time series models described in Section 2.3. 

#### 2.2.1. Correlations and Time-Lag Identification

Correlations between each type of antibiotic usage and its corresponding antibiotic resistance rate were examined. In line with the criteria employed by Ryu et al. (2018) and Hsu et al. (2010), we selected antibiotics for which the correlation with resistance rates had a *p*-value less than 0.05 and R-squared value greater than 0.3; these antibiotics were further analyzed in our time series studies presented in Section 2.3 [12,27]. If no antibiotics met those criteria, then antibiotics whose *p*-values are simply less than 0.05 were selected. Because the usage of antibiotics might not immediately lead to the emergence of antibiotic resistance, cross-correlation analysis was conducted to identify potential ‘time-lags’ between when an antibiotic is used and when the emergence of antibiotic resistance can be observed. For the cross-correlation analysis, monthly lags of up to 1 year in both directions were applied to the antibiotic resistance series. The most likely time-lag at which antibiotic usage is significantly associated with antibiotic resistance rates was then determined. This determination was based on the strength of the correlation coefficient values at each given time-lag and its statistical significance [12,28]. 

#### 2.2.2. Stepwise Linear Regression and Time-Lag Identification

The stepwise linear regression was performed by using all the antibiotics as predictor variables; the stepAIC() function in R 3.6.2 was used to execute stepwise model selection, in which the model with the lowest Akaike Information Criterion (AIC) was picked. From this reduced model, the top independent variables were chosen, defined as those with *p*-values less than 0.05. Cross-correlation analysis was conducted on those chosen antibiotics to identify the most likely time-lag at which antibiotic usage is significantly associated with antibiotic resistance rates. Independent variables whose *p*-values are less than 0.05 and the adjusted R-squared value of the model are reported in the Section 3. 

#### 2.2.3. Recursive Feature Selection with the Random Forest Algorithm and Time-Lag Identification

The most important features were iteratively identified and retained, based on their contribution to the model as measured by their percent increase in mean squared error (%IncMSE) [29]. This recursive feature selection was performed using the random forest algorithm with the rfe() function of the ‘caret’ library in R 3.6.2. Subsequently, cross-correlation analysis was conducted on the selected features, in this case antibiotics, to identify the most likely time-lag at which antibiotic usage is significantly associated with antibiotic resistance rates.

### 2.3. Time Series Modeling

The auto.arima() function in R 3.6.2 was used to build ARIMA models. Neural networks were built using the nnetar() function in R 3.6.2. This function builds simple neural networks with a single hidden layer and takes in non-seasonal lags as inputs. The number of inputs and the number of nodes in the hidden layer were modified to identify the most optimal model, whose performance was measured by MAE and RMSE. Finally, random forest algorithm regression time series models were built using the RandomForest package in R 3.6.2 [19]. Parameter tuning for the random forest algorithm focused on the ‘mtry’ parameter, which represents the number of variables considered for splitting at each tree node. We utilized the ‘caret’ package’s ‘train’ function with a time-slice approach, employing a ‘trainControl’ method with a ‘timeslice’ option. This method simulated a realistic forecasting scenario by keeping a fixed validation window at the end of the training dataset, spanning the forecast horizon of twelve months. The tuning grid was established with three ‘mtry’ values: the total number of predictors, a third of this number, and the square root of the number of predictors, following common random forest heuristics. 

The top predictors, i.e., antibiotics, selected in Section 2 by the correlation analysis, stepwise linear regression, and recursive feature elimination process were used as exogenous regressors to the ARIMA, neural network, and random forest time-series models [12,27].

### 2.4. Assessing Performance and Making Predictions

The performance of the time-series models was measured by MAE and RMSE [15,30,31]. The best performing model that showed the least signs of overfitting or underfitting, as measured by the ratio of performance on training data to test data, was used to predict the antibiotic resistance rates over the next 12 months, from January 2020 to December 2020, based on the expected usage of the identified antibiotics.

## 3. Results

### 3.1. Correlations and Time-Lag Identification

**Figure 1:**
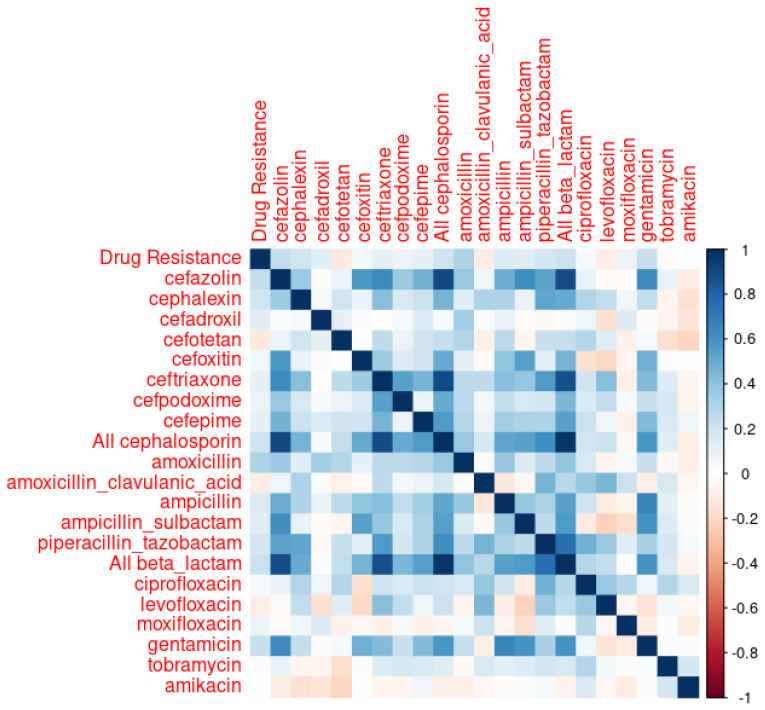
Correlations Between ‘Drug Resistance’ and Antibiotic Usage.

Correlations between each type of antibiotic use and corresponding antibiotic resistance rates for the train data are depicted in Figure 1 and tabulated in Table 2. As Table 2 shows, both amoxicillin and cefazolin met one of the selection criteria defined in Section 2: their correlations with drug resistance had *p*-values less than 0.05. However, none of the R-squared values in Table 2 were greater than 0.3. In the cross-correlation analyses, amoxicillin exhibited a 5-month lag with a cross correlation value of 0.322 (*p* < 0.05), and cefazolin showed no lag. 

### 3.2. Stepwise Linear Regression and Time-Lag Identification

As shown in Table 3, from the stepwise linear regression of all predictor variables, amoxicillin, cefotetan, and cephalexin were identified as antibiotics significantly associated with antibiotic resistance, as indicated by their *p*-values less than 0.05 (i.e., 0.00289, 0.01918, and 0.03301, respectively). Approximately 16.4% variations associated with resistant *E. coli* can be explained by cephalexin, amoxicillin, cefotetan, and amoxicillin/clavulanic acid usage. No significant cross-correlation time-lag was found for both cefotetan and cephalexin [28]. 

### 3.3. Recursive Feature Selection with the Random Forest Algorithm and Time-Lag Identification

**Figure 2:**
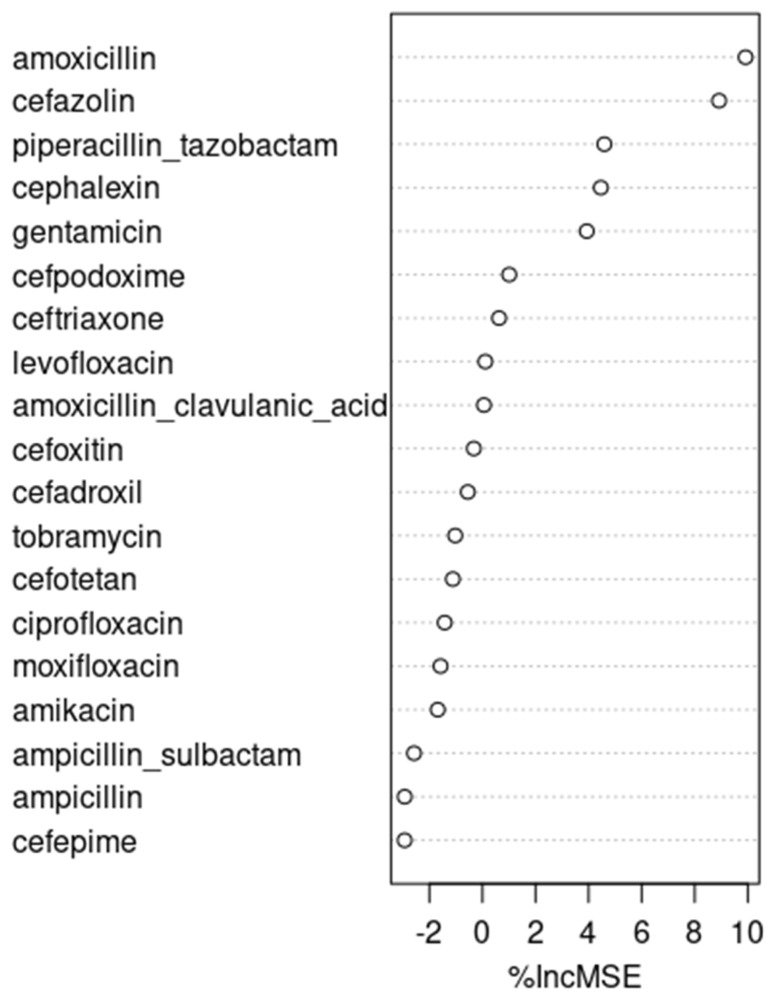
Recursive feature selection with random forest algorithm.

Recursive feature selection with the random forest algorithm reported amoxicillin, cefazolin, and piperacillin/tazobactam as the top three factors associated with antibiotic resistance, as shown in Figure 2. Because amoxicillin and cefazolin already were picked from correlation analyses and stepwise linear regression, piperacillin/tazobactam was picked as one of our candidates. Under cross-correlation analysis, no significant time-lag was identified for piperacillin/tazobactam. 

### 3.4. Time Series Modeling

RMSE and MAE of ARIMA, neural networks, and the random forest time series models, with cephalexin, amoxicillin, cefazolin, cefotetan, and piperacillin/tazobactam as exogenous regressors at corresponding time-lags identified by cross correlation analyses, are in Table 4, Table 5, Table 6, Table 7 and Table 8 as follows:

Although the random forest model with cefazolin (no lag) as an exogenous regressor performed the best in the test dataset, the underfitting observed in the training data set disqualifies this model as the optimal representation of reality. The ARIMA model with cefazolin (no lag), followed by the ARIMA model with cephalexin (with no lag), performed the best in terms of RMSE and MAE values across both the train and test data sets. 

Therefore, using the ARIMA model with cefazolin as an external regressor, and assuming the same rate of cefazolin usage as in 2019, we concluded that the predicted rate of resistant *E. coli* infections in 2020 was 6.2%, with its 95% prediction interval being 0.9% to 11.5%. When a 50% reduction is made in cefazolin usage, the predicted rate of resistant *E. coli* in 2020 was 4.52%, with its 95% prediction interval being −0.1% to 9.8%. With a 25% reduction in cefazolin usage, the predicted rate of resistant *E. Coli* in 2020 was 5.36%, with its 95% prediction interval being 0% to 10.7%.

## 4. Discussion

It is interesting to note that cefazolin and cephalexin, both first-generation cephalosporins with similar spectrums of activity, were found to be closely associated with the resistant *E. coli* infections we observed. Cefazolin is frequently used in perioperative settings and in long-term bacteremia treatments, representing 11.2% of all antibiotics usage observed in our data set. On the other hand, cephalexin is usually used in outpatient settings, and represents only 0.57% of the observed inpatient antibiotics usage. Given the relatively narrower spectrums of activity of cefazolin and cephalexin compared to other antibiotics, targeting these two antibiotics for ASP interventions is often challenging. We encounter this scenario because antibiotics stewardship programs try to limit the use of unnecessarily broad-spectrum antibiotics under the assumption that they exert Darwinian pressure, generating antibiotics-resistant microorganisms. 

At the same time, although piperacillin/tazobactam, a broad-spectrum antibiotic, did not perform as well as cefazolin and cephalexin, it performed fairly well in the ARIMA and neural network time series models, ranking right after cefazolin and cephalexin. Had we picked piperacillin/tazobactam to make our predictions, the predicted rate of resistant *E. coli* in 2020, according to the ARIMA model, and assuming the same rate of piperacillin/tazobactam usage as in 2019, was 5.52%, with its 95% prediction interval being 0.14% to 10.9%. When a 50% reduction is made in piperacillin/tazobactam usage, the predicted rate of resistant *E. coli* in 2020 was 4.31%, with its 95% prediction interval being −1.1% to 9.7%. With a 25% reduction in piperacillin/tazobactam usage, the predicted rate of resistant *E. coli* was 4.91%, with its 95% prediction interval being −0.47% to 10.3%. 

While one might be tempted to build a time series model with both cefazolin and piperacillin/tazobactam—excluding cephalexin because it is rarely used in inpatient settings—such a model does not perform better than cefazolin alone, as shown in Table 9:

Although the variance inflation factor (VIF)—a measure to quantify the severity of multicollinearity in an ordinary least squares regression analysis—value for cefazolin and piperacillin/tazobactam is 1.39, which is not particularly concerning for collinearity, the performance of a linear model combining these two factors is less than satisfactory. Specifically, the adjusted R-value for a model with piperacillin/tazobactam and cefazolin together is 0.03477, which is lower than the 0.0446 adjusted R-value for a model with cefazolin alone. This lower adjusted R-value suggests that the combined model does not explain the variance in the dependent variable as well as the cefazolin-alone model does. Moreover, the moderate increase in the adjusted R-value from a piperacillin/tazobactam-only model (which is 0.02) to a piperacillin/tazobactam and cefazolin combination model (which is 0.03477) does not seem sufficient to justify the time series model that includes both piperacillin/tazobactam and cefazolin as regressors.

An interesting analysis can be made with amoxicillin and cephalexin, whose inpatient use is negligible at 0.15% and 0.57%, respectively. Those figures might underestimate the actual use of amoxicillin and cephalexin, as those two oral antibiotics are frequently prescribed just before patient discharge. Although patients might receive those medications for only one or two days during their inpatient stay, the therapy usually extends much longer post-discharge. Those two antibiotics became notable because of the stepwise linear regression shown in Table 3, in which the usage of four antibiotics amoxicillin, cefotetan, cephalexin, and amoxicillin/clavulanic acid explain a non-negligible amount—more specifically, 16.4%—of variations associated with resistant *E. coli*. Of those, the use of amoxicillin, cefotetan, and cephalexin demonstrated significant correlations with the emergence of antibiotic resistance, with respective *p*-values of 0.00289, 0.01918, and 0.03301. After excluding cefotetan, an intravenous antibiotic that is rarely (i.e., 0.032%) used, and then primarily in perioperative settings, we built a linear model of the two oral antibiotics, amoxicillin and cephalexin. That linear model explains 9.5% of variations associated with observed resistant *E. coli* cases, with a *p*-value of 0.01369. Therefore, it might be worthwhile to consider those two antibiotics as external regressors when building time-series models.

The performance of those models, tabulated in Table 10 (with amoxicillin at lag 5 and cephalexin at lag 0), is not particularly outstanding. However, the best of them, the ARIMA model, was used to make predictions in recognition of the potential significance of those two antibiotics, as indicated by the strength of their linear model. 

Using the ARIMA model with amoxicillin at lag 5 and cephalexin at lag 0 as external regressors, we concluded that the predicted rate of resistant *E. coli* infections in 2020, assuming the same rate of amoxicillin and cephalexin usage as in 2019, was 5.5%, with a 95% prediction interval of 0.31% to 10.7%. When a 50% reduction is made in amoxicillin and cephalexin usage, the predicted rate of resistant *E. coli* in 2020 was 4.56%, with a 95% prediction interval of −0.61% to 9.72%. With a 25% reduction in amoxicillin and cephalexin usage, the predicted rate of resistant *E. coli* in 2020 was 5.0%, with a 95% prediction interval of −0.15% to 10.2%.

Overall, our analyses suggest that while making interventions for cefazolin might not be practical in light of its relatively narrow spectrum, and the focus of many ASP programs that primarily target the use of broad-spectrum antibiotics, judiciously reducing the use of piperacillin/tazobactam in inpatient settings and the use of amoxicillin and cephalexin at discharge could lower *E. coli*-resistant infection rates [6,13]. Both time series models with either piperacillin/tazobactam or amoxicillin and cephalexin showed about a 0.5% drop in antibiotic resistance rates, from approximately 5.5 to 5%, when the use of either group of antibiotics was reduced by 25%. In 2019, there were 1009 total *E. coli* infection cases at the Kaiser Vacaville facility in the U.S., and this number on average increased by 81 cases every year. Extrapolating, we can expect 1090 cases in 2020. Therefore, a 0.5% drop in antibiotic resistance rates achieved via an ASP program could translate to approximately $80,000 per year in savings for *E. coli* infections alone, because each Gram-negative resistant infection extends the LOS of a patient by 5 days on average [9] and the average cost of inpatient stay per day is $2883 [14]. Considering many other Gram-negative infections caused by other organisms, such as Klebsiella, Pseudomonas, and Acinetobacter, we could anticipate multiples of $80,000 in savings per year per hospital. 

It is noteworthy that the ARIMA model outperformed both the neural network and the random forest models. This is in line with a systematic review published in 2019, which found a lack of evidence to support the superiority of machine learning algorithms over logistic regression in clinical prediction models [32]. At the same time, one of the reasons the neural network model did not perform as well as we initially expected might be attributed to the limitations associated with the rather simple nnetar() function in R that we used.

The results of this study are based on longitudinal data collected from one Kaiser Permanente facility located in a somewhat rural region with relatively low population migration. In the future, a broader study could be conducted using data from multiple facilities. Additionally, while the performance of the statistical and machine learning models was evaluated against the test dataset from the final year in our data set, 2019, a follow-up study could be carried out to validate the predictions made for 2020 using actual data from that year. However, conducting such a study is currently beyond our scope, as this study was approved by Kaiser Permanente for a pharmacist resident project in 2021, utilizing data spanning from 2013 to 2019. 

Finally, while this study identifies significant correlations between antibiotic usage and the emergence of resistant *E. coli* infections, it is crucial to recognize that these findings do not establish direct causation. The inherent limitations of observational data in confirming causal relationships necessitate caution in interpretation. Consequently, our results highlight the need for further research, including experimental or more comprehensive longitudinal studies, to rigorously explore causality. 

## 5. Conclusions

While cefazolin might be most significantly associated with drug-resistant *E. coli* infections, its relatively narrow spectrum makes it a difficult intervention target. Although piperacillin/tazobactam, a broad-spectrum antibiotic, did not perform as well as cefazolin in our time series models, it performed fairly sufficiently. Even though amoxicillin and cephalexin are not frequently used in hospitals, they seem to have a significant association with the rates of drug-resistant *E. coli.* Practical interventions for amoxicillin and cephalexin can be made at the point of discharge. For follow-up studies, one could develop models for other Gram-negative infections, and furthermore include outpatient antibiotics use data. Moreover, as discussed earlier, conducting a follow-up study with data from multiple Kaiser Permanente facilities and from the year 2020 could be beneficial. This research provides a foundational structure that encourages ASP clinicians to integrate statistical and machine-learning methodologies, leveraging local data to develop targeted interventions. 

## Figures and Tables

**Table 1:** Inclusion and Exclusion Criteria.

Inclusion Criteria
All *E. coli* cultures (urine, blood, abscess, wound, genital, and GI abscess).
Admission to KP Vacaville between 1 April 2013 and 31 December 2019.
Administration of antibiotics, which include cefazolin; cephalexin; cefadroxil; cefotetan; cefoxitin; ceftriaxone; cefpodoxime; cefepime; piperacillin/tazobactam; amoxicillin; amoxicillin/clavulanic acid; ampicillin; ampicillin/sulbactam; moxifloxacin; levofloxacin; ciprofloxacin; gentamicin; tobramycin; and amikacin. Daily doses were reported as days of therapy per 1000 patient days (DOT/1000 patient days).
For patients with multiple episodes of antibiotic resistant (indicated by resistance to ceftriaxone) *E. coli* infections, only the first episode was included [26].
**Exclusion Criterion**
Patients not meeting inclusion criteria.

**Table 2:** Correlations between ‘Drug Resistance’ and Antibiotics Usage.

	Antibiotics	Correlation	*p*-Value
Drug Resistance	amoxicillin	0.313	0.009
Drug Resistance	cefazolin	0.2423	0.0450
Drug Resistance	gentamicin	0.225	0.063
Drug Resistance	all beta lactams	0.218	0.0715
Drug Resistance	all cephalosporins	0.206	0.0888
Drug Resistance	cephalexin	0.193	0.113
Drug Resistance	piperacillin/tazobactam	0.186	0.127
Drug Resistance	ampicillin	0.146	0.232
Drug Resistance	ampicillin/sulbactam	0.1396	0.253
Drug Resistance	cefadroxil	0.126	0.302
Drug Resistance	ceftriaxone	0.115	0.345
Drug Resistance	cefepime	0.103	0.398
Drug Resistance	cefpodoxime	0.0835	0.495
Drug Resistance	moxifloxacin	0.083	0.499
Drug Resistance	cefoxitin	0.057	0.644
Drug Resistance	ciprofloxacin	0.046	0.709
Drug Resistance	amikacin	0.000	0.999
Drug Resistance	tobramycin	−0.018	0.885
Drug Resistance	amoxicillin/clavulanic acid	−0.094	0.440
Drug Resistance	levofloxacin	−0.097	0.428
Drug Resistance	cefotetan	−0.113	0.356

**Table 3:** Stepwise Linear Regression results.

Coefficients:	Estimate	Std. Error	t Value	Pr (>|t|)
(Intercept)	5.095 × 10^−2^	1.087 × 10^−2^	4.685	0.000 ***
cephalexin	9.309 × 10^−7^	4.272 × 10^−7^	2.179	0.033 *
cefotetan	−1.341 × 10^−6^	5.581 × 10^−7^	−2.403	0.019 *
amoxicillin	2.004 × 10^−6^	6.469 × 10^−7^	3.098	0.003 **
amoxicillin/clavulanic acid	−4.869 × 10^−7^	3.024 × 10^−7^	−1.610	0.112
Signif. codes: 0 ‘***’ 0.001 ‘**’ 0.01 ‘*’ 0.05				
Residual standard error: 0.025 on 64 degrees of freedom				
Multiple R-squared: 0.213, Adjusted R-squared: 0.164				
F-statistic: 4.339 on 4 and 64 DF, *p*-value: 0.004				

**Table 4:** Performance of time-series models with cephalexin as an external regressor.

Cephalexin with No Lag	RMSE (Train)	MAE (Train)	RMSE (Test)	MAE (Test)
ARIMA	0.027	0.0223	0.030	0.024
Neural Network	0.023	0.019	0.032	0.027
Random Forest	0.051	0.041	0.040	0.035

**Table 5:** Performance of time series models with amoxicillin with a 5-month lag as an external regressor.

Amoxicillin with 5 Months Lag	RMSE (Train)	MAE (Train)	RMSE (Test)	MAE (Test)
ARIMA	0.027	0.022	0.033	0.026
Neural Network	0.022	0.018	0.035	0.029
Random Forest	0.060	0.048	0.042	0.036

**Table 6:** Performance of time series models with cefazolin as an external regressor.

Cefazolin with No Lag	RMSE (Train)	MAE (Train)	RMSE (Test)	MAE (Test)
ARIMA	0.027	0.022	0.027	0.024
Neural Network	0.024	0.019	0.029	0.025
Random Forest	0.046	0.037	0.019	0.017

**Table 7:** Performance of time series models with cefotetan an external regressor.

Cefotetan with No Lag	RMSE (Train)	MAE (Train)	RMSE (Test)	MAE (Test)
ARIMA	0.029	0.022	0.034	0.027
Neural Network	0.025	0.019	0.034	0.027
Random Forest	0.054	0.043	0.051	0.043

**Table 8:** Performance of time series models with piperacillin/tazobactam as an external regressor.

Piperacillin/Tazobactam with No Lag	RMSE (Train)	MAE (Train)	RMSE (Test)	MAE (Test)
ARIMA	0.027	0.022	0.031	0.026
Neural Network	0.025	0.020	0.030	0.025
Random Forest	0.048	0.039	0.056	0.050

**Table 9:** Performance of time series models with cefazolin and piperacillin/tazobactam as external regressors.

Cefazolin and Piperacillin/Tazobactam, No Lags	RMSE (Train)	MAE (Train)	RMSE (Test)	MAE (Test)
ARIMA	0.027	0.022	0.027	0.025
Neural Network	0.022	0.018	0.030	0.027
Random Forest	0.044	0.034	0.033	0.025

**Table 10:** Performance of time-series models with amoxicillin (lag 5) and cephalexin as external regressors.

Amoxicillin Lag 5 and Cephalexin, No Lags	RMSE (Train)	MAE (Train)	RMSE (Test)	MAE (Test)
ARIMA	0.027	0.022	0.033	0.026
Neural Network	0.021	0.017	0.034	0.028
Random Forest	0.053	0.043	0.050	0.045

## Data Availability

The data that support the findings of this study are available from Kaiser Permanente but restrictions apply to the availability of these data, which were used under license for the current study, and so are not publicly available. The data are, however, available from the authors upon reasonable request and with permission of Kaiser Permanente.
